# Perceived Threat Associated with Police Officers and Black Men Predicts Support for Policing Policy Reform

**DOI:** 10.3389/fpsyg.2016.01057

**Published:** 2016-07-12

**Authors:** Allison L. Skinner, Ingrid J. Haas

**Affiliations:** ^1^Department of Psychology, University of WashingtonSeattle, WA, USA; ^2^Department of Political Science and Center for Brain, Biology, and Behavior, University of Nebraska-LincolnLincoln, NE, USA

**Keywords:** threat, race, police, policy, prejudice, discrimination

## Abstract

Racial disparities in policing and recent high-profile incidents resulting in the deaths of Black men have ignited a national debate on policing policies. Given evidence that both police officers and Black men may be associated with threat, we examined the impact of perceived threat on support for reformed policing policies. Across three studies we found correlational evidence that perceiving police officers as threatening predicts increased support for reformed policing practices (e.g., limiting the use of lethal force and matching police force demographics to those of the community). In contrast, perceiving Black men as threatening predicted reduced support for policing policy reform. Perceived threat also predicted willingness to sign a petition calling for police reform. Experimental evidence indicated that priming participants to associate Black men with threat could also reduce support for policing policy reform, and this effect was moderated by internal motivation to respond without prejudice. Priming participants to associate police officers with threat did not increase support for policing policy reform. Results indicate that resistance to policing policy reform is associated with perceiving Black men as threatening. Moreover, findings suggest that publicizing racially charged police encounters, which may conjure associations between Black men and threat, could reduce support for policing policy reform.

## Introduction

A series of high-profile police altercations with Black men, including those resulting in the deaths of Michael Brown and Eric Garner in 2014, have ignited a national debate on policing policies in the United States (Davis, 2015). In response, the “Black Lives Matter” movement has risen to prominence, with many calling for policing policy reform and increased police oversight. Given that this issue is both salient and important, The Supreme Court took up the issue of excessive force in June of 2015 (Kingsley v. Hendrickson, 2015). President Obama also responded by forming a task force to investigate policing practices, calling on police departments to make changes consistent with the task force's recommendations—such as matching police force demographics to those of the communities they serve and independent investigations of lethal force incidents (Davis, 2015). Meanwhile, the public is fairly divided on policing policy reform. For example, data from a large demographically representative opinion poll indicates that 52% of Americans support police force demographic matching and 35% think that it is unnecessary (Frankovic, 2015). The goal of the current research was to examine some of the psychological factors underlying attitudes about policing policy.

## Threat associated with police officers

One potentially important factor that may help explain policing policy positions is the extent to which the actors involved are perceived as threatening. Threat is particularly relevant given that both police officers and Black men are associated with threat (e.g., Duncan, 1976; Tyler, 2011). Police officers in the U.S. rely on the threat of physical force to motivate compliance (Tyler, 2011; Tyler et al., 2015). This approach may translate into perceptions of the police as threatening, especially among those who find themselves frequent targets of unwarranted police attention (e.g., racial minorities). Indeed, at least among young men in the U.S., there is a tendency to expect negative interactions with police (Najdowski et al., 2015). Moreover, media is an integral component of experience in contemporary society (Liu and Crank, 2010), therefore even those that do not have personal experience with police are exposed to police-related media. Across all races, people who are exposed to media reports of police abuse and those who report personal or vicarious experience with police abuse perceive higher rates of police misconduct (Weitzer and Tuch, 2004). Furthermore, given that negative experiences tend to be more salient (Weitzer and Tuch, 2004), recent media coverage has likely led to an increase in negative attitudes toward police. Gallup poll data from 2011 to 2014 indicates that 14% of Americans reported having very little or no confidence in police (Newport, 2014). Yet, more recent YouGov poll data suggests that trust and confidence in police dipped from 2014 to 2015. As of April 2015, 39% of U.S. adults indicated that they had little or no trust in police officers nationwide (Moore, 2015). Thus, those who perceive the police as particularly threatening may be more supportive of policing policies that limit police power.

## Threat associated with black men

There is considerable evidence that in the U.S. Black men are stereotyped as hostile and aggressive, and tend to be associated with threat (Duncan, 1976; Devine and Elliot, 1995; Eberhardt et al., 2004). For example, priming participants with the concept of crime brings Black faces to mind, whereas priming participants with Black faces increases the cognitive accessibility of threatening objects (e.g., guns; Eberhardt et al., 2004). Moreover, people tend to misidentify objects held by Black targets (relative to White targets) as threatening and are quicker to “shoot” Black vs. White targets in laboratory simulations (Correll et al., 2011), an effect that is particularly pronounced for Black male targets (Plant et al., 2011). Thus, it is conceivable that those who perceive Black men as particularly threatening would be more supportive of policing policies that will be restrictive of Black men.

Given recent calls for policy changes aimed at curtailing excessive use of force and reducing racial disparities in police altercations, we aimed to investigate the role of threat in public support for reformed policing policy. Specifically, we examined the relationship between perceived threat associated with both police officers and Black men on support for reformed policing policies. We hypothesized that perceiving police officers as threatening would predict support for reformed policing practices that increase police oversight and accountability. Yet, perceiving Black men as threatening was expected to predict reduced support for reformed policing practices. By investigating the relationship between threat associations and policy positions in a time of intense societal debate we hoped to capture the nature of these relationships within the contemporary cultural context.

## Overview of the current studies

Studies 1a and 1b were framed within the context of the Michael Brown shooting in Ferguson, Missouri, in August 2014 and aimed to measure (1) the relationship between perceived threat associated with police and policing policy positions and (2) the relationship between perceived threat associated with Black men and policing policy positions. In Study 2, we tested whether experimentally-manipulated threat associations with police officers would increase support for policing policy reform. In Study 3, we tested whether experimentally-manipulated threat associations with Black men would reduce support for policing policy reform. Finally, in Study 4 we used a more subtle form of associative conditioning to manipulate threat associations with police officers or Black men. Study 4 also included a measure of behavioral intentions, to assess participants' willingness to take action in support of policing policy reform.

## Study 1A methods

### Participants

Student participants (*N* = 224) were recruited from a participant pool at a large public university in a conservative state in the Midwestern United States. Eight participants did not provide complete data and were dropped from the study, leaving a sample of 216 participants (52.8% men). Four participants chose not to provide complete demographic data, thus all demographic data is only for the participants for which demographic data was provided. The mean age of participants was 20.18 years (*SD* = 3.49). Most participants identified as White (93.1%) and the remainder of participants self-identified as Asian (2.3%), Black (1.4%), Native American (0.5%), or another race (2.8%), with 5.6% of participants identifying as Hispanic or Latino. Politically, participants identified as moderate to somewhat conservative (*M* = 4.47, *SD* = 1.54), on a scale from “very liberal” (*1*) to “very conservative” (*7*). Participants were compensated with partial course credit for participating in this study. Data collection began approximately 8 weeks after Michael Brown's death (on September 30, 2014) and continued for nearly 7 weeks (through December 1, 2014). Data collection concluded once we exceeded the predetermined sample size of 200.

### Materials and procedure

This research was conducted in compliance with the ethical standards for participant treatment set by the APA and was approved by the Institutional Review Board for the University of Nebraska-Lincoln. Participants accessed the survey online through Qualtrics. After providing written informed consent participants were reminded of the Michael Brown shooting using the following passage: “The shooting of Michael Brown occurred on August 9, 2014, in Ferguson, Missouri, a suburb of St. Louis. Michael Brown, a Black 18-year-old, was fatally shot by a White law enforcement officer, Darren Wilson. The resultant protests and civil disorder received considerable attention in the United States and abroad.” Then, in two separate items, participants rated the extent to which they felt threatened by “law enforcement officers” and “Black men” *as a result of the Michael Brown shooting*, on a five-point scale ranging from “a lot less threatened” to “a lot more threatened.” Next, participants responded to the four policing policy reform items listed below. These items referred to specific policing policies, rather than a constellation of traits or beliefs that would be expected to reliably relate to one another, thus we did not expect these items to systematically covary. Moreover, given the ongoing dialogue about these issues in the media during the time data was collected, we anticipated that the relationships between these items might vary over time. Thus, we did not intend to combine these items into a single scale. To confirm that items were not highly related we conducted reliability analyses, which confirmed that internal reliability was low (Cronbach's Alpha = 0.43). Additional exploratory items were included in our data collection for Studies 1a and 1b, but because they are not directly related to policing policy (the focus of the current paper) they have not been included.

1. In order to increase law enforcement accountability, some police departments have begun requiring officers to wear on-body cameras. To what extent do you agree with this type of policy? *Answered on a 5-point scale from “agree completely” to “disagree completely.”*
2. How do you believe law enforcement officers who use deadly force on civilians should be treated? *Answered on a 5-point scale from “they should undergo a lot less scrutiny than civilians” to “they should undergo a lot more scrutiny than civilians.”*
3. Do you believe the racial demographics of a police force should be representative of the racial demographics in the community they serve?
  - Police force racial demographics should not be influenced by the racial demographics of the community.
  - Police force racial demographics should be influenced a little bit by the racial demographics of the community.
  - Police force racial demographics should match racial demographics of the community.
4. When do you believe it is appropriate for law enforcement officers to use deadly force? SELECT ALL THAT APPLY
  - When someone is using deadly force on them.
  - When they believe someone will use deadly force on them.
  - When someone is physically injuring them.
  - When they believe someone will physically injure them.
  - When someone is non-cooperative.
  - When they believe someone will be non-cooperative.
  - If someone has committed a crime.
  - When they believe someone will be committing a crime.
  - If someone is committing a crime.

Before exiting the survey participants provided demographic information, then they were thanked and credited for their participation.

### Results

Table 1 presents the percentage of participants that deemed lethal force acceptable in each of the presented circumstances. For analysis we calculated a sum of these items, such that participants who believed lethal force was appropriate under all circumstances scored a *9* and participants that who believed lethal force was appropriate under no circumstances scored a 0. Participants reported feeling somewhat more threatened by both groups (as a result of the Michael Brown shooting), although they were significantly more threatened by police officers (*M* = 3.36, *SD* = 0.62) than Black men [*M* = 3.12, *SD* = 0.37, *t*_(215)_ = 4.71, *p* < 0.001]. The two threat items showed a weak negative correlation [*r*_(216)_ = −0.14, *p* = 0.038], indicating a small inverse relationship between threat associated with police officers and threat associated with Black men.

**Table 1:** **Table represents the percentage of participants (in descending order) responding in the affirmative that it is appropriate for police officers to use deadly force in each circumstance**.

When someone is using deadly force on an officer	97.20%
When someone is physically injuring an officer	64.50%
When an officer believes someone will use deadly force on them	52.10%
When someone is committing a crime.	24.90%
When an officer believes someone will physically injure them	16.10%
If someone has committed a crime	8.30%
When someone is non-cooperative	4.60%
When an officer believes someone will be committing a crime	2.80%
When an officer believes someone will be non-cooperative	0.90%

A multivariate regression analysis was conducted to examine the role of threat associated with police officers and threat associated with Black men in predicting support for the policing policy items. Multivariate analysis results indicated that threat associated with police officers [*F*_(4, 209)_ = 7.59, *p* < 0.001, $\eta_{p}^{2}$ = 0.13] and threat associated with Black men [*F*_(4, 209)_ = 4.33, *p* < 0.001, $\eta_{p}^{2}$ = 0.08] significantly predicted support for policing policy reform. Moreover, inspection of the parameter estimates indicated that all associations were in the expected direction. Greater threat associations with police officers predicted increased support for policing policy reform and greater threat associations with Black men predicted decreased support for policing policy reform. Next, we repeated this analysis controlling for race, ethnicity, age, and gender. Multivariate analysis results indicated that after controlling for demographic factors, threat associated with police officers [*F*_(4, 195)_ = 6.05, *p* < 0.001, $\eta_{p}^{2}$ = 0.11] and threat associated with Black men [*F*_(4, 195)_ = 3.99, *p* = 0.004, $\eta_{p}^{2}$ = 0.08] still significantly predicted support for policing policy reform. Table 2 provides the full results for each dependent measure and adjusted *R*^2^ for models (a) including only demographic covariates, (b) only the threat measures, and (c) the threat measures controlling for demographic covariates.

**Table 2:** **Results of Study 1a regression analysis using threat associated with police officers and Black men to predict support for policing policy reform items**.

	**Police officers**					**Black men**					**Adjusted *R*^2^**
	**β**	***B***	***SE***	***p***	**$\eta_{p}^{2}$**	**β**	***B***	***SE***	***p***	**$\eta_{p}^{2}$**	
Scrutiny (a)											0.005
(b)	0.30	0.50	0.11	<0.001	0.10^*^	−0.15	−0.40	0.18	0.023	0.02^*^	0.119
(c)	0.29	0.48	0.11	<0.001	0.08^*^	−0.14	−0.38	0.18	0.041	0.02^*^	0.111
Cameras (a)											0.085
(b)	−0.06	−0.10	0.11	0.354	0.00	0.03	0.08	0.18	0.681	0.00	−0.004
(c)	−0.07	−0.12	0.11	0.287	0.01	0.03	0.09	0.18	0.595	0.00	0.083
Demographic matching (a)											0.034
(b)	0.29	0.26	0.08	0.001	0.05^*^	−0.06	−0.12	0.12	0.317	0.01	0.053
(c)	0.26	0.23	0.08	0.004	0.04^*^	−0.07	−0.13	0.13	0.290	0.01	0.074
Lethal force justified (a)											0.010
(b)	−0.13	−0.30	0.15	0.051	0.02	0.24	0.89	0.25	<0.001	0.06^*^	0.074
(c)	−0.12	−0.28	0.16	0.077	0.02	0.24	0.90	0.26	<0.001	0.06^*^	0.081

*Model 1 (a) includes only the demographic covariates (age, race, ethnicity, and gender), model 2 (b) contains only the threat measures, and model 3 (c) includes threat measures, controlling for demographic covariates. Statistically significant (p < 0.05) effects are indicated by an asterisk*.

## Study 1B

In Study 1b we aimed to replicate Study 1a findings in a more demographically representative sample. To do this we recruited participants from Amazon's *Mechanical Turk*, an online workforce of hundreds of thousands of people who complete tasks in exchange for monetary compensation. Empirical investigation of data produced by the *Mechanical Turk* workforce indicates that samples are more representative than typical college samples and are at least equally reliable (Buhrmester et al., 2011). We hypothesized that the same general pattern of results would emerge in a sample of adult community members.

### Method

#### Participants

Adult community members (*N* = 278) were recruited from Amazon Mechanical Turk in the United States. Nine participants did not provide complete data and were dropped from the study, leaving a sample of 269 participants (48.4% men). Seventeen participants chose not to provide complete demographic data, thus all demographic data is only for the participants for which demographic data was provided. The mean age of participants was 36.34 years (*SD* = 11.83). Most participants identified as White (74.3%) and the remainder of participants self-identified as Black (8.9%), Asian (7.1%), Native American (1.1%), or another race (2.6%), with 5.6% of participants identifying as Hispanic or Latino. Politically, participants identified as moderate to somewhat liberal (*M* = 3.30, *SD* = 1.68), on a scale from “very liberal” (*1*) to “very conservative” (*7*). Participants were compensated $1.00 for participating in this study. Data was collected between October 1 and October 6, 2014, approximately 2 months after the Michael Brown shooting. Data collection concluded when we exceeded the predetermined (based on allocated funding) sample size of 250. Materials and procedures for Study 2 were identical to those used for Study 1.

### Results

The percentage of participants that deemed lethal force acceptable in each of the presented circumstances in Study 1b was quite similar to Study 1a (see Supplemental Materials). Yet, for *physically injuring an officer* (54.60%) and *committing a crime* (11.90%) our community sample was less accepting of police officers' use of deadly force. Participants reported feeling somewhat more threatened by both groups (as a result of the Michael Brown shooting) although they were significantly more threatened by police officers (*M* = 3.42, *SD* = 0.70) than Black men [*M* = 3.10, *SD* = 0.57, *t*_(268)_ = 5.73, *p* < 0.001]. Our two threat items were uncorrelated in this sample [*r*_(267)_ = −0.08, *p* = 0.22], indicating that they were tapping into unrelated threat constructs, rather than a sense of generalized threat.

**Figure 1:**
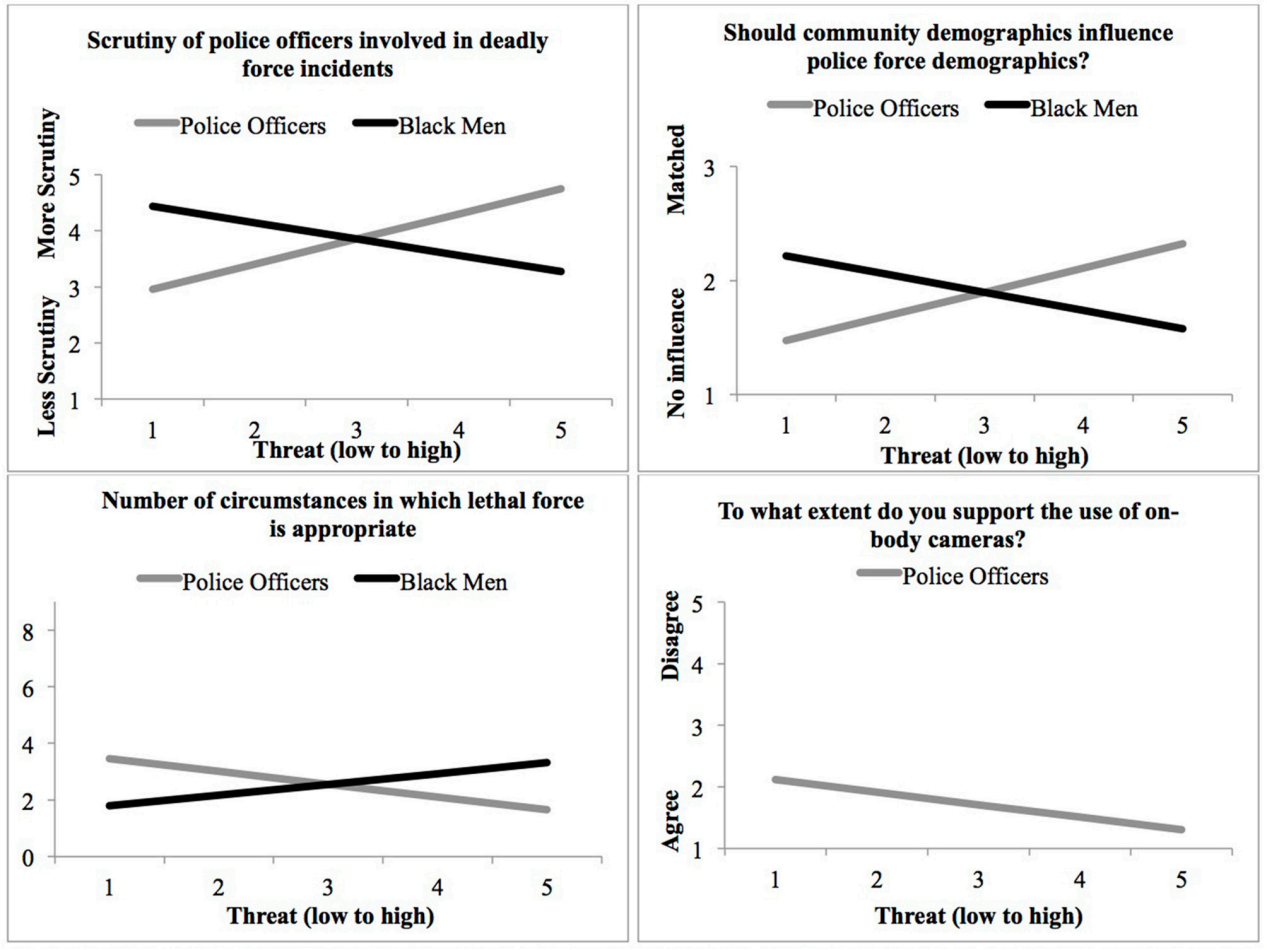
**Panels depict the significant relationships between threat associated with police officers and Black men and each of the four policy outcome measures from Study 1b**.

Multivariate regression analysis results indicated that threat associated with police officers [*F*_(4, 263)_ = 12.14, *p* < 0.001, $\eta_{p}^{2}$ = 0.16] and threat associated with Black men [*F*_(4, 263)_ = 3.92, *p* = 0.004, $\eta_{p}^{2}$ = 0.06] significantly predicted support for policing policy reform. Moreover, inspection of the parameter estimates indicated that all associations were in the expected direction. Greater threat associations with police officers predicted increased support for policing policy reform and greater threat associations with Black men predicted decreased support for policing policy reform. Next, we repeated this analysis controlling for race, ethnicity, age, and gender. Multivariate analysis results indicated that after controlling for demographic factors, threat associated with police officers [*F*_(4, 238)_ = 13.04, *p* < 0.001, $\eta_{p}^{2}$ = 0.18] and threat associated with Black men [*F*_(4, 238)_ = 3.93, *p* = 0.004, $\eta_{p}^{2}$ = 0.06] still significantly predicted support for policing policy reform. Table 3 provides the full results for each dependent measure and adjusted *R*^2^ for models (a) including only demographic covariates, (b) only the threat measures, and (c) the threat measures controlling for demographic covariates. Figure 1 presents model predicted support for each policing policy reform item as a function of perceived threat ratings.

**Table 3:** **Results of Study 1b regression analysis using threat associated with police officers and Black men to predict support for policing policy reform items**.

	**Police officers**					**Black men**					**Adjusted *R*^2^**
	**β**	***B***	***SE***	***p***	**$\eta_{p}^{2}$**	**β**	***B***	***SE***	***p***	**$\eta_{p}^{2}$**	
Scrutiny (a)											−0.019
(b)	0.31	0.44	0.08	<0.001	0.10^*^	−0.16	−0.28	0.10	0.005	0.03^*^	0.120
(c)	0.34	0.49	0.09	<0.001	0.12^*^	−0.18	−0.32	0.11	0.004	0.03^*^	0.129
Cameras (a)											−0.012
(b)	−0.19	−40.21	0.07	0.002	0.04^*^	0.04	0.05	0.08	0.526	0.00	0.031
(c)	−0.19	−0.21	0.07	0.003	0.04^*^	0.04	0.06	0.09	0.481	0.00	0.020
Demographic matching (a)											−0.002
(b)	0.20	0.21	0.06	0.001	0.04^*^	−0.13	−0.17	0.08	0.034	0.02^*^	0.053
(c)	0.23	0.25	0.07	<0.001	0.06^*^	−0.16	−0.21	0.09	0.016	0.02^*^	0.073
Lethal force justified (a)											0.044
(b)	−0.24	−0.46	0.11	<0.001	0.06^*^	0.17	0.40	0.14	0.003	0.03^*^	0.087
(c)	−0.24	−0.46	0.12	<0.001	0.06^*^	0.16	0.38	0.15	0.010	0.03^*^	0.125

*Model 1 (a) includes only the demographic covariates (age, race, ethnicity, and gender), model 2 (b) contains only the threat measures, and model 3 (c) includes threat measures, controlling for demographic covariates. Statistically significant (p < 0.05) effects are indicated by an asterisk*.

### Study 1 discussion

Studies 1a and 1b provide evidence that perceived threat associated with Black men and perceived threat associated with police officers predict support for policing policy reform. Consistent with our hypotheses, participants who felt more threatened by police officers as a result of the Michael Brown shooting reported *more* support for policing policy reform. These findings suggest that those who feel threatened by police are motivated to limit police power by reforming policing practices. Participants who felt more threatened by Black men as a result of the Michael Brown shooting reported *less* support for policing policy reform. Across both studies, feeling threatened by Black men predicted broader acceptance of lethal force (i.e., endorsement of lethal force under a broader set of circumstances), less support for scrutiny of officers that use lethal force, and less support for demographic matching. These findings suggest that those who perceive Black men as particularly threatening tend to be more supportive of policing policies that are restrictive of Black men.

## Study 2

Studies 1a and 1b provided evidence that perceiving police officers as threatening predicts policing policy positions, but were correlational in nature and cannot establish the existence of a causal relationship. The goal of Study 2 was to examine whether changes in threat associations can produce changes in policing policy positions. We hypothesized that priming participants with threatening images of police officers would cause support for policing policy reform to increase.

### Method

#### Participants

Student participants (*N* = 126) were recruited from a participant pool at a large public university in a conservative state in the Midwestern United States. Three participants did not provide complete data and were dropped from the study, leaving a sample of 123 participants (52% men). The mean age of participants was 20.31 years (*SD* = 2.58). Most participants identified as White (83.7%) and the remainder of participants self-identified as Asian (6.5%), Black (2.4%), Native American (1.6%), or another race (5.7%), with 4.1% of participants identifying as Hispanic or Latino. Politically, participants identified as moderate (*M* = 3.90, *SD* = 1.73), on a scale from “very liberal” (*1*) to “very conservative” (*7*), though one participant did not report political ideology. Participants were compensated with partial course credit for participating in this study. Data was collected April 6–24, 2015. Data collection concluded when the participant pool closed for the academic semester.

### Materials and procedure

This research was conducted in compliance with the ethical standards for participant treatment set by the APA and was approved by the Institutional Review Board for the University of Nebraska-Lincoln. Participants accessed the survey online through Qualtrics. After providing written informed consent participants were randomly assigned to one of two prime conditions associating police officers with threat or no threat. Prime condition was subtly manipulated via images inconspicuously presented at the top of each screen of the survey (photos are available from the authors upon request). In the threat condition participants viewed groups of menacing looking police officers, standing off against unseen protesters. None of the images depicted weapons, violence, or destruction of property. Control (non-threatening) images depicted groups of police officers with happy or neutral facial expressions in neutral contexts (e.g., casually walking down the street).

Participants responded to the four policing policy reform items used in Studies 1a and 1b, which were presented directly below the images. Each page of the survey included the same series of images presented as a banner across the top of the screen. Only one question was presented on each page to ensure that the prime images were always in full view while participants completed each item. After completing the primary dependent measures, we asked participants to rate how threatening they found the stimulus images to be on a scale from *0* (not at all) to *100* (completely). Before exiting the survey participants provided demographic information, then they were thanked and credited for participation.

### Results

First, we confirmed that images in the threat condition (*M* = 62.96, *SE* = 2.94) were rated as significantly more threatening than images in the control condition [*M* = 17.25, *SE* = 3.31; *t*_(119)_ = 10.14, *p* < 0.001]. The percentage of participants that deemed lethal force acceptable in each of the presented circumstances in Study 2 was quite similar to Studies 1a and 1b (see Supplemental Materials).

Multivariate analysis of variance results indicated that threat condition did not significantly predict support for policing policy reform [*F*_(4, 118)_ = 1.21, *p* = 0.309, $\eta_{p}^{2}$ = 0.04]. Next, we repeated this analysis controlling for race, ethnicity, age, and gender. Multivariate analysis results indicated that after controlling for demographic factors, threat condition still did not significantly predict support for policing policy reform [*F*_(4, 108)_ = 1.54, *p* = 0.196, $\eta_{p}^{2}$ = 0.05]. Table 4 provides the full results for each dependent measure and adjusted *R*^2^ for models (a) including only demographic covariates, (b) only the threat manipulation, and (c) the threat manipulation controlling for demographic covariates. Due to a failure of random assignment, sample size was not equivalent across conditions, resulting in 26 additional participants in the threat condition. All effects remained the same if the additional participants were removed.

**Table 4:** **Results of Study 2 multivariate analysis of variance using manipulated threat associated with police officers to predict support for policing policy reform items**.

	**Police threat prime**			**Adjusted *R*^2^**
	***F***	***p*^2^**	**$\eta_{p}^{2}$**	
Scrutiny (a)				0.036
(b)	0.38	0.541	0.00	−0.005
(c)	0.21	0.645	0.00	0.029
Cameras (a)				−0.012
(b)	3.01	0.085	0.02	0.016
(c)	3.21	0.076	0.03	0.008
Demo matching (a)				0.011
(b)	0.30	0.578	0.00	−0.006
(c)	0.46	0.500	0.00	0.006
Lethal Force (a)				−0.011
(b)	0.88	0.351	0.01	−0.001
(c)	2.25	0.137	0.02	0.000

*Model 1 (a) includes only the demographic covariates (age, race, ethnicity, and gender), model 2 (b) contains only the threat manipulation, and model 3 (c) includes the threat manipulation, controlling for demographic covariates*.

### Study 2 discussion

Study 2 failed to support our hypothesis, that priming threat associated with police officers would increase support for policing policy reform. Overall we found no evidence that experimentally manipulating threat associated with police officers increases support for policing policy reform. It is possible that viewing threatening images of police officers may not have increased the type of personally relevant feelings of threat necessary to effect policing policy positions. In other words, participants may have perceived the images to be threatening, but because they do not see police officers as a personally relevant threat they were not motivated to reform policing policy.

## Study 3

Study 3 was designed to test whether priming threat associations with Black men would reduce support for policing policy reform. Threat associations with Black men and threat associations with police officers are conceptually independent, therefore the lack of policy position change in Study 2 is conceptually independent from the question examined in Study 3. We hypothesized that priming participants with threatening images of Black men would cause support for policing policy reform to decrease.

There is considerable evidence of anti-Black bias and stereotyping in the U.S. that can be observed throughout the lifespan (i.e., as early as childhood; Baron and Banaji, 2006)—thus, priming the association between Black men and threat has the potential to activate preexisting racial biases. To assess the role of underlying racial biases we also included a measure of motivation to respond without prejudice. Previous research shows that those with high internal motivation to respond without prejudice (IMS) tend to score low in racial bias (Plant and Devine, 1998; Hausmann and Ryan, 2004), thus we hypothesized that internal motivation to respond without prejudice would moderate the effect of prime on policing policy reform. Specifically, we predicted that those with high IMS (i.e., lower racial bias) would tend to support policing policy reform, but that this tendency would be lessened in the threat condition. Meanwhile, we predicted that those with low IMS (i.e., higher racial bias) would tend to object to policing policy reform and that this tendency would be exacerbated in the threat condition.

### Method

#### Participants

Student participants (*N* = 128, 57% men) were recruited from a participant pool at a large public university in a conservative state in the Midwestern United States. The mean age of participants was 19.86 years (*SD* = 1.54). Most participants identified as White (83.6%) and the remainder of participants self-identified as Asian (7.8%), Black (3.1%), or another race (5.5%), with 3.1% of participants identifying as Hispanic or Latino. Politically, participants identified as moderate (*M* = 3.90, *SD* = 1.66), on a scale from “very liberal” (*1*) to “very conservative” (*7*). Participants were compensated with partial course credit for participating in this study. Data was collected April 6–24, 2015. Data for studies 2 and 3 were collected simultaneously, thus participants who signed up to participate were randomly assigned to Study 2 (threat or control) or Study 3 (threat or control). Data collection concluded when the participant pool closed for the academic semester.

### Materials and procedure

This research was conducted in compliance with the ethical standards for participant treatment set by the APA and was approved by the Institutional Review Board for the University of Nebraska-Lincoln. Participants accessed the survey online through Qualtrics. After providing written informed consent participants were randomly assigned to one of two prime conditions, associating Black men with threat or no threat. Images were presented in the same fashion as Study 2 (see Appendix for photos in Supplementary Materials). In the threat condition participants viewed groups of threatening-looking Black male protesters, though none of the images depicted weapons, violence, or destruction of property. Control (non-threatening) images depicted groups of Black men with happy or neutral facial expressions in neutral contexts (e.g., casually standing outdoors). All study procedures were the same as Study 2 except that before exiting the survey participants completed the IMS scale (Plant and Devine, 1998). The IMS scale includes items such as “I am personally motivated by my beliefs to be non-prejudiced toward Black people.” The IMS scale was highly reliable (Cronbach's Alpha = 0.85).

### Results

First, we confirmed that images in the threat condition (*M* = 45.71, *SE* = 3.36) were rated as significantly more threatening than images in the control condition [*M* = 15.73, *SE* = 3.05; *t*_(126)_ = 6.60, *p* < 0.001]. The percentage of participants who deemed lethal force acceptable in each of the presented circumstances in Study 3 was similar to the previous Studies (see Supplemental Material).

Multivariate analysis of variance results indicated that threat condition did not significantly predict support for policing policy reform [*F*_(4, 123)_ = 1.67, *p* = 0.161, $\eta_{p}^{2}$ = 0.05]. Next, to examine the effect of racial bias on support for policing policy reform, we added mean centered IMS (*M* = 7.21, *SD* = 1.49) to the model. Results of the multivariate analysis of covariance including IMS indicated that IMS significantly predicted support for policing policy reform [*F*_(4, 122)_ = 3.29, *p* = 0.014, $\eta_{p}^{2}$ = 0.10].

Finally, we added the threat condition by IMS interaction to the model. Results of the multivariate analysis of covariance including the IMS interaction indicated that the IMS by threat condition interaction significantly predicted support for policing policy reform [*F*_(4, 121)_ = 3.37, *p* = 0.012, $\eta_{p}^{2}$ = 0.10]. Next, we repeated this analysis controlling for race, ethnicity, age, and gender. Multivariate analysis of covariance results indicated that after controlling for demographic factors, the IMS by threat condition interaction still significantly predicted support for policing policy reform [*F*_(4, 113)_ = 3.53, *p* = 0.009, $\eta_{p}^{2}$ = 0.11]. Table 5 provides the full results for each dependent measure and adjusted *R*^2^ for each of the following models (a) including only the demographic covariates (age, race, ethnicity, and gender), (b) containing only the threat manipulation, (c) including the threat manipulation and the main effect of IMS, (d) including the threat manipulation, the main effect of IMS, and their interaction, and (e) including all the predictors, controlling for demographic covariates.

**Table 5:** **Results of Study 3 multivariate analysis of covariance using manipulated threat associated with Black men to predict support for policing policy reform items**.

	**Black men threat prime**			**IMS**			**Prime X IMS**			**Adjusted *R*^2^**
	***F***	***p***	**$\eta_{p}^{2}$**	***F***	***p***	**$\eta_{p}^{2}$**	***F***	***p***	**$\eta_{p}^{2}$**	
Scrutiny (a)										0.023
(b)	2.04	0.156	0.02							0.008
(c)				9.30	0.003	0.07^*^				0.067
(d)							1.79	0.183	0.01	0.073
(e)							1.01	0.317	0.01	0.122
Cameras (a)										0.078
(b)	0.93	0.338	0.01							−0.001
(c)				2.13	0.147	0.02				0.008
(d)							3.55	0.062	0.03	0.028
(e)							8.42	0.004	0.07^*^	0.128
Demo matching (a)										0.010
(b)	5.00	0.027	0.04^*^							0.031
(c)				4.89	0.029	0.04^*^				0.060
(d)							8.73	0.004	0.07^*^	0.114
(e)							5.96	0.016	0.05^*^	0.111
Lethal Force (a)										0.045
(b)	0.02	0.887	0.00							−0.008
(c)				1.35	0.248	0.01				−0.005
(d)							1.73	0.191	0.01	0.001
(e)							0.46	0.501	0.00	0.039

*Model 1 (a) includes only the demographic covariates (age, race, ethnicity, and gender), model 2 (b) contains only the threat manipulation, model 3 (c) includes the threat manipulation and the main effect of IMS, model 4 (d) includes the threat manipulation, the main effect of IMS, and their interaction, and Model 5 (e) includes all the predictors from model 4, controlling for demographic covariates. Statistically significant (p < 0.05) effects are indicated by an asterisk*.

Follow-up analyses examining the multivariate effects of IMS in each experimental condition indicated that in the control condition there was a significant effect of IMS on support for policing policy reform, [*F*_(4, 113)_ = 6.25, *p* < 0.001, $\eta_{p}^{2}$ = 0.18]. Thus, in the control condition, as IMS increased (i.e., as racial bias decreased), support for policing policy reform increased. In contrast, in the Black threat condition there was no effect of IMS on support for policing policy reform, [*F*_(4, 113)_ = 1.40, *p* = 0.239, $\eta_{p}^{2}$ = 0.05]. Follow-up analyses examining the multivariate effects at high (1 SD above the mean) and low (1 SD below the mean) levels of IMS indicated that at high levels of IMS there was a significant effect of threat manipulation on support for policing policy reform, [*F*_(4, 113)_ = 4.13, *p* = 0.004, $\eta_{p}^{2}$ = 0.13]. At high levels of IMS (i.e., low levels of racial bias) participants in the threat condition were less supportive of policing policy reform than participants in the control condition. In contrast, at low levels of IMS (i.e., high levels of racial bias) participants in the threat condition were no less supportive of policing policy reform than participants in the control condition, [*F*_(4, 113)_ = 0.88, *p* = 0.476, $\eta_{p}^{2}$ = 0.03]. Thus, although participants with low racial bias were more supportive of policing policy reform under control conditions, when primed with Black-threat associations participants with low racial bias were no more supportive of policing policy reform than participants with high racial bias.

### Study 3 discussion

Study 3 provided evidence for the hypothesized link between prejudice and policing policy reform. Consistent with previous literature indicating that those with high IMS show the lowest levels of racial bias (Plant and Devine, 1998; Hausmann and Ryan, 2004), participants with high IMS were most supportive of policing policy reform. Moreover, we found evidence of an interaction between threat conditioning and motivation to respond without prejudice. Priming threat associations with Black men influenced policing policy positions among high IMS participants, such that high IMS participants who were primed with Black threat were less supportive of policing policy reform than those in the control condition. These findings are consistent with our predictions that priming threat associations with Black men would decrease support for policing policy reform among those with low racial bias. We also predicted that priming threat associations with Black men would further reduce support for policing policy reform among those with high racial bias. Yet, we found no evidence to support this; those with low IMS did not vary their policing policy support as a function of condition. The current evidence suggests that priming threat associations with Black men only influences the policing policy positions of those with high IMS. Thus, although low prejudice people tend to be more supportive of some policing policy reforms under control conditions, when they are primed to associate Black men with threat they are no more supportive of policing policy reforms than high prejudice people.

## Study 4

Taken together Studies 2 and 3 suggest that experimentally manipulating threat associations with Black men may influence policing policy positions, whereas manipulating threat associations with police officers may not have a significant influence on policing policy positions. It is possible, however, that although participants rated the images of police in Study 2 as threatening, they did not perceive images of police officers facing off against (unseen) protesters as personally relevant. In other words, for participants that do not engage in police protests (which is likely most of them), the Study 2 manipulation may not have induced the personally relevant threat necessary to motivate policing policy position shifts. To address this issue, in Study 4 we employed a threat association conditioning paradigm, in which threatening images were paired with images of police officers or Black men. This experimental approach works by causing participants to misattribute emotions (e.g., threat) elicited by an unconditioned stimulus to a subsequently presented conditioned stimulus (e.g., police officers or Black men; Olson and Fazio, 2006). Thus, the effectiveness of this manipulation is not contingent upon participants' pre-existing views related to police protests or the policing policy debate. A second goal of study 4 was to examine a potential mediator of the relationship between Black threat associations and policing policy positions—fear of crime. Specifically, we aimed to test whether associating threat with Black men increases fear of crime, which leads to resistance to policing policy reform. Our third goal was to determine whether this threat manipulation would impact behavioral intentions, in this case, willingness to sign a petition calling for police reform. Our fourth and final goal was to reexamine the relationship between existing threat associations and policing policy positions outside the context of the Michael Brown case.

### Method

#### Participants

Adult community members (*N* = 267) were recruited from Amazon Mechanical Turk in the United States. Four participants did not provide complete data and were dropped from the study, leaving a sample of 262 participants (63.40% men). The mean age of participants was 33.69 years (*SD* = 10.32). Most participants identified as White (77%) and the remainder of participants self-identified as Asian (11%), Black (7%), or another race (5%), with 6% of participants identifying as Hispanic or Latino. Politically, participants identified as moderate to somewhat liberal (*M* = 3.11, *SD* = 1.59), on a scale from “very liberal” (*1*) to “very conservative” (*7*). Participants were compensated $1.00 for participating in this study. Data was collected between August 17 and August 23, 2015. Data collection concluded when we exceeded the predetermined (based on allocated funding) sample size of 250.

### Materials and procedure

This research was conducted in compliance with the ethical standards for participant treatment set by the APA and was approved by the Institutional Review Board for the University of Nebraska-Lincoln. Participants were told they would be completing a cognitive memory and reaction time task and then a separate survey on their political attitudes. Participants accessed the survey online through Qualtrics and, after providing written informed consent, they were randomly assigned to one of three prime conditions: police officers paired with threat, Black men paired with threat, or general threat (not paired with either group). We used classical conditioning (Olson and Fazio, 2006) to create these threat associations, such that participants were exposed to eight critical pairings of threatening images (e.g., snakes, ferocious dogs) followed by prime images (i.e., Black men or police officers), embedded within a series of 60 images. Pilot testing indicated that the threatening images induced fear, as participants used words such as *scared, frightened, uneasy*, and *anxious* to describe the emotions elicited by the images. All threat images and several of the distractor images (e.g., neutral/positive images of animals, cars, landscapes, people) were drawn from the International Affective Picture System (IAPS; Lang et al., 2008)^1^; all additional images were drawn from various online sources. Prime images depicted Black men and police officers displaying neutral to positive facial expressions in neutral contexts. Participants saw the same images in all three conditions, presented one at a time in the center of the screen. However, the order of images systematically varied, such that in the police threat condition, threatening images always immediately preceded images of police officers whereas in the in the Black threat condition, threatening images always immediately preceded images of Black men. In the general threat (control) condition, order of presentation of threatening images was entirely random.

As a cover story, and to distract participants from the prime, we informed them that the task was a test of memory (for specific target categories) and reaction time. Participants were presented with two target categories (animals and landscapes) and instructed to identify images representing those target categories as quickly as possible when they appeared. On trials in which a target was present participants were instructed to press the “q” key and on trials in which a target was absent they were instructed to press the “p” key. Images remained onscreen until participants responded. Before beginning the main task participants completed a total of 12 practice trials in which they first practiced identifying animals (6 trials) and then practiced identifying landscapes (6 trials) before moving on to the main task, in which they were instructed to identify both targets. On each of the 12 practice trials participants received an error message if they failed to identify a target or incorrectly identified a non-target—emphasizing the importance of correctly categorizing targets. Throughout the priming task instructions were visible to participants, reminding them to press “q” for targets and “p” for non-targets and to respond as quickly as possible.

After completing the priming task, participants were asked to complete a demographic questionnaire and thanked for their participation in the cognitive task. Next, they were prompted to complete a brief survey on their political attitudes. First, participants completed a series of four items designed to assess their fear of crime. Fear of crime items were modeled off those used by Hetey and Eberhardt (2014) and included “how worried are you about crime in your community?” and “to what extent do you agree that crime is increasing in your community?,” and two identical items inquiring about crime in the United States. All four items were answered on a scale from *1* (not at all) to *6* (extremely), and formed a reliable scale (Cronbach's Alpha = 0.86).

Next, participants responded to the four policing policy reform items used in the previous studies. Then, to assess behavioral intentions, we presented participants with a mock online petition calling on the U.S. Federal Government to “demand national change to protect citizens and communities from police violence and misconduct.” They were then asked whether, given the opportunity, they would be willing to sign that petition. Finally, we asked participants to rate how threatening they find police officers and Black men on a scale from *0* (not at all) to *100* (completely). Following these items participants were thanked and credited for participation.

### Results

#### Analysis approach and explicit threat ratings

First we tested whether our threat manipulation impacted explicit threat ratings of Black men and police officers. Results indicated that condition did not impact explicit threat ratings for Black men or police (*p*s > 0.35). As with Studies 1a and 1b, participants rated police officers (*M* = 39.98, *SD* = 31.34) as significantly more threatening than Black men [*M* = 32.65, *SD* = 29.42, *t*_(261)_ = −3.20, *p* = 0.002]. The two threat rating items showed a fairly small but significant positive correlation [*r*_(262)_ = 0.26, *p* < 0.001], indicating that those who felt threatened by police officers also tended to feel threatened by Black men. Given that condition did not impact explicit threat rating and evidence from our previous studies indicating that threat rating is an important individual difference predictor, we included threat ratings (rescaled to range from 0 to 10 and mean centered) as predictors in all of the following models. Thus, the multivariate model included reference coded effects of the Black threat prime, the police threat prime, mean centered Black threat rating, and mean centered police threat rating.

#### Fear of crime

Participants indicated that crime is worse in the United States as a whole (*M* = 3.72, *SD* = 1.30; *M* = 3.60, *SD* = 1.47) than it is in their own communities (*M* = 2.85, *SD* = 1.29; *M* = 2.74, *SD* = 1.44). Results of the full model indicated that Black threat rating significantly predicted fear of crime, such that as threat rating increased fear of crime increased [*B* = 0.10, *SE* = 0.02, *F*_(1, 257)_ = 17.09, *p* < 0.001, $\eta_{p}^{2}$ = 0.06]. None of the other effects were statistically significant (all *p*s > 0.10). The effect of Black threat rating remained statistically significant after controlling for participant demographic factors (race, ethnicity, age, and gender), *F*_(1, 249)_ = 15.19, *p* < 0.001, $\eta_{p}^{2}$ = 0.05.

#### Policing policy positions

Again, the percentage of participants that deemed lethal force acceptable in each of the presented circumstances was quite similar to the previous studies (see Supplemental Materials). Multivariate results are reported below and Table 6 provides the full results for each dependent measure and adjusted *R*^2^ for models (a) including only demographic covariates, (b) only the threat manipulation and threat ratings, and (c) the threat manipulation controlling for demographic covariates.

**Table 6:** **Results of Study 4 multivariate analysis of covariance using reference coded effects of the Black threat prime, the police threat prime, mean centered Black threat rating, and mean centered police threat rating to predict support for policing policy reform items**.

	**Black men threat prime**			**Police threat prime**			**Black men threat rating**			**Police threat rating**			**Adjusted *R*^2^**
	***F***	***p***	**$\eta_{p}^{2}$**	***F***	***p***	**$\eta_{p}^{2}$**	***F***	***p***	**$\eta_{p}^{2}$**	***F***	***p***	**$\eta_{p}^{2}$**	
Scrutiny (a)													0.011
(b)	1.82	0.179	0.01	2.16	0.142	0.01	12.34	0.001	0.05^*^	46.76	<0.001	0.15^*^	0.156
(c)	0.82	0.366	0.00	1.43	0.232	0.01	13.78	<0.001	0.05^*^	42.70	<0.001	0.15^*^	0.170
Cameras (a)													−0.008
(b)	1.50	0.221	0.01	0.23	0.631	0.00	7.47	0.007	0.03^*^	8.17	0.005	0.03^*^	0.037
(c)	1.99	0.160	0.01	0.18	0.673	0.00	7.77	0.006	0.03^*^	8.72	0.003	0.03^*^	0.029
Demo matching (a)													0.055
(b)	1.59	0.208	0.01	1.13	0.288	0.00	4.83	0.029	0.02^*^	7.09	0.008	0.03^*^	0.027
(c)	0.69	0.406	0.00	0.99	0.322	0.00	4.83	0.029	0.02^*^	8.34	0.004	0.03^*^	0.088
Lethal Force (a)													0.009
(b)	1.90	0.169	0.01	1.11	0.293	0.00	40.79	<0.001	0.14^*^	46.29	<0.001	0.15^*^	0.206
(c)	2.39	0.124	0.01	1.72	0.191	0.01	40.01	<0.001	0.14^*^	43.05	<0.001	0.15^*^	0.214

*Model 1 (a) includes only the demographic covariates (age, race, ethnicity, and gender), model 2 (b) contains only the threat primes and measures, and model 3 (c) includes the threat manipulation, controlling for demographic covariates. Statistically significant (p < 0.05) effects are indicated by an asterisk*.

##### Experimental manipulation

Multivariate analysis results indicated that neither the police threat manipulation [*F*_(4, 254)_ = 1.76, *p* = 0.137] nor the Black threat manipulation condition [*F*_(4, 254)_ = 1.28, *p* = 0.279] significantly influenced policing policy positions. Next, we repeated this analysis controlling for race, ethnicity, age, and gender. Multivariate analysis results indicated that after controlling for demographic factors, the police threat manipulation still did not significantly predict support for policing policy reform [*F*_(4, 246)_ = 1.49, *p* = 0.204] nor did the Black threat manipulation [*F*_(4, 246)_ = 1.23, *p* = 0.297].

##### Threat associated with police officers

Multivariate analysis results indicated that the self-reported threat associated with police officers predicted support for policing policy reform [*F*_(4, 254)_ = 20.84, *p* < 0.001]. The more threatened participants reported feeling by police officers the more they supported reforms to policing policy. Next, we repeated this analysis controlling for race, ethnicity, age, and gender. Multivariate analysis results indicated that after controlling for demographic factors, the effect of threat associated with police officers on support for policing policy reform remained significant [*F*_(4, 246)_ = 19.59, *p* < 0.001].

##### Threat Associated with Black Men

Multivariate analysis results indicated that the self-reported threat associated with Black men predicted support for policing policy reform [*F*_(4, 254)_ = 12.82, *p* < 0.001]. The more threatened participants reported feeling by Black men the less they supported reforms to policing policy. Next, we repeated this analysis controlling for race, ethnicity, age, and gender. Multivariate analysis results indicated that after controlling for demographic factors, the effect of threat associated with Black men on support for policing policy reform remained significant [*F*_(4, 246)_ = 12.97, *p* < 0.001].

#### Behavioral intentions: willingness to sign a petition for policing policy reform

**Figure 2:**
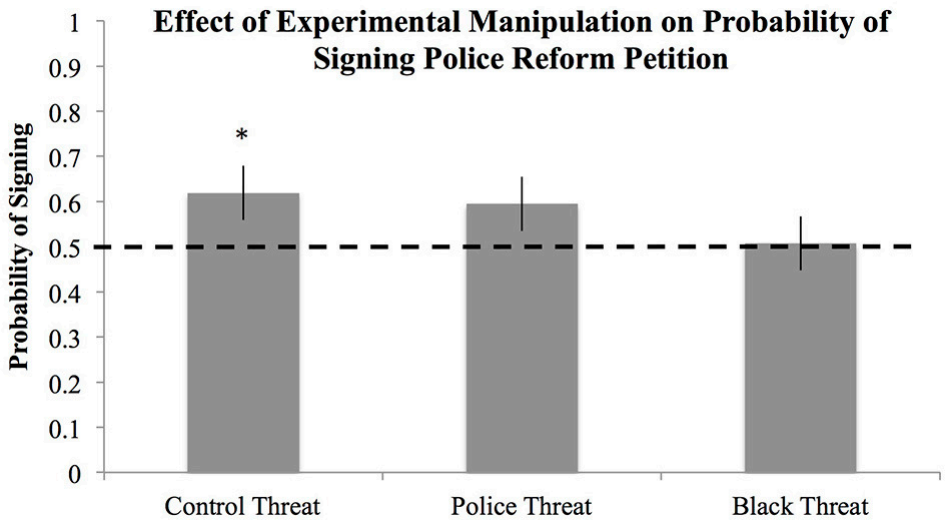
**Probability of signing a petition in support of police reform as a function of threat condition**. ^*^Indicates significant deviation from chance (*p* < 0.50), error bars represent standard errors.

We used logistic regression to examine the effects of condition and explicit threat ratings on willingness to sign the petition. First, we tested a logistic model including only demographic covariates (Adjusted *R*^2^ = 0.08). Next, we tested a model including only the threat manipulation and threat ratings (Adjusted *R*^2^ = 0.32). Results indicated that although the threat conditions did not significantly differ from one another (*B*s < |0.29|, *p*s > 0.17), participants in the control condition were significantly more likely to sign the petition than chance (*Probability* = 0.62, *SE* = 0.06, *p* = 0.036, 95% CI [0.51, 0.72]; See Figure 2). Participants in the Black threat condition were not significantly more likely to sign the petition than chance (*Probability* = 0.51, *SE* = 0.06, *p* = 0.902, 95% CI [0.39, 0.62]), nor were the participants in the police threat condition (*Probability* = 0.59, *SE* = 0.06, *p* = 0.110, 95% CI [0.48, 0.70]). Lastly, we tested a model including the threat manipulation, and threat ratings, controlling for demographic factors (Adjusted *R*^2^ = 0.36)^2^.

Explicit threat ratings of both police officers and Black men also predicted the probability of signing the petition. The more threatened participants felt by police officers the greater the probability that they would sign the petition (*B* = 0.25, *SE* = 0.03, *p* < 0.001, 95% CI [0.18, 0.31]) and the more threatened participants felt by Black men the lower the probability that they would sign the petition (*B* = −0.13, *SE* = 0.03, *p* < 0.001, 95% CI [−0.20, −0.06]). Results from the model controlling for participant demographic factors was nearly identical. The more threatened participants felt by police officers the greater the probability that they would sign the petition (*B* = 0.27, *SE* = 0.04, *p* < 0.001, 95% CI [0.19, 0.34]) and the more threatened participants felt by Black men the lower the probability that they would sign the petition (*B* = −0.16, *SE* = 0.04, *p* < 0.001, 95% CI [−0.23, −0.08]).

#### Examining fear of crime as a mediator

To investigate whether fear of crime might mediate the relationship between self-reported threat associations and policing policy positions we conducted a multivariate regression analysis using fear of crime to predict policing policy positions. Results indicated that fear of crime significantly predicted support for policing policy reform [*F*_(4, 257)_ = 2.68, *p* = 0.032]. Yet, univariate analyses indicated that fear of crime only significantly predicted positions on one policy. The more fearful participants felt about crime, the more instances in which lethal force was deemed acceptable (*B* = 0.21, *SE* = 0.07, *p* = 0.004, $\eta_{p}^{2}$ = 0.03). Fear of crime was not significantly related to support for scrutiny of officers that use lethal force (*B* = −0.10, *SE* = 0.05, *p* = 0.071, $\eta_{p}^{2}$ = 0.01), demographic matching (*B* = −0.07, *SE* = 0.04, *p* = 0.081, $\eta_{p}^{2}$ = 0.01), or support for the use of on-body cameras (*B* = 0.02, *SE* = 0.04, *p* = 0.558, $\eta_{p}^{2}$ = 0.00). Results from the model controlling for participant demographic factors were similar, although some effects increased in statistical significance. Multivariate results indicated that fear of crime still significantly predicted support for policing policy reform [*F*_(4, 249)_ = 3.22, *p* = 0.013]. Univariate analyses indicated that the more fearful participants felt about crime, the more instances in which lethal force was deemed acceptable (*B* = 0.21, *SE* = 0.07, *p* = 0.005, $\eta_{p}^{2}$ = 0.03). Fear of crime also predicted a significant increase in support for demographic matching (*B* = −0.10, *SE* = 0.04, *p* = 0.012, $\eta_{p}^{2}$ = 0.02). Fear of crime still did not predict support for the use of on-body cameras (*B* = 0.01, *SE* = 0.04, *p* = 0.816, $\eta_{p}^{2}$ = 0.00) or scrutiny of officers that use lethal force (*B* = −0.09, *SE* = 0.06, *p* = 0.113, $\eta_{p}^{2}$ = 0.01).

Next, for each of the two outcomes in which fear of crime was a significant predictor, we examined whether fear of crime was still a significant predictor when included in the larger model with threat associations. Fear of crime was no longer a significant predictor of the number of incidents in which deadly force is deemed acceptable when included in the larger model (*B* = 0.12, *SE* = 0.07, *p* = 0.082). Yet, fear of crime was still a statistically significant predictor of demographic matching (*B* = −0.09, *SE* = 0.04, *p* = 0.026). Moreover, the statistical significance of threat associated with Black men dropped to non-significant (*B* = −0.03, *SE* = 0.02, *p* = 0.108) when fear of crime was included in the model. To examine whether fear of crime significantly mediates the effect of perceived threat associated with Black men on support for demographic matching, we conducted a mediation analysis using the PROCESS MACRO (SAS 9.3). Results using 1000 Bootstrap samples indicated that the direct effect of threat associated with Black men on support for demographic matching was not significant (*B* = −0.03, *SE* = 0.02, *p* = 0.081, 95% CI [−0.06, 0.00]). The confidence interval for the indirect effect did not contain 0, indicating a significant indirect effect through fear of crime (*B* = −0.01, *SE* = 0.00, 95% CI [−0.02, −0.00]).

#### Study 4 discussion

The primary goal of Study 4 was to address the design limitations of Studies 2 and 3 by using a context independent manipulation of threat (i.e., conditioning paradigm). We hypothesized that using this type of approach, threat associations with Black men *and* threat associations with police would influence policing policy positions. Yet, results indicated that the more subtle threat conditioning manipulation did not significantly influence policing policy positions. Relative to the control condition all means in the Black threat condition were in the expected direction (except for the use of force variable), but none of them reached statistical significance. The conditioning manipulation also did not influence fear of crime. Yet, the conditioning manipulation did impact behavioral intentions, such that for those in the control condition the probability of signing the petition calling for police reform was significantly greater than chance. In contrast, in the Black threat condition the probability of signing the petition was at chance. Probability of signing the petition also did not differ from chance in the police threat condition, yet inspection of the estimated probabilities of signing the petition indicate that the police threat condition (0.59) is much closer to that of the control condition (0.62) than that of the Black threat condition (0.51). Thus, we found some evidence that the threat association manipulation influenced behavioral intentions—but not policing policy positions. At this point it is unclear to what extent threat associations with Black men and police *cause* changes in policing policy positions. It is possible that within the larger societal context, in which participants were being bombarded with media messages about the policing policy debate, it may take more than a brief conditioning task to change policing policy positions. It is also possible that the effect of the conditioning procedure was weakened by the online presentation of stimuli (e.g., variability in loading time, external distractions), such that a more highly controlled lab setting might have produced stronger effects.

Study 4 also provided an opportunity to reexamine the relationship between self-reported threat associations and policing policy positions, outside the context of the Michael Brown case. Consistent with our previous findings, as threat associated with Black men increased, participants showed reduced support for policing policy reform. On the other hand, as threat associated with police officers increased, participants showed increasing support for policing policy reform. Overall, Study 4 findings indicate that threat associations with police officers and Black men predict policing policy positions—and that this relationship is not limited to the context of the Michael Brown shooting. Threat associations also predicted behavioral intentions, such that feeling threatened by Black men decreased the probability of signing the petition for police reform and feeling threatened by police officers increased the probability of signing the petition.

We found that feeling threatened by Black men increased fear of crime, and that fear of crime mediated the effect of Black-threat associations on support for demographic matching. Yet fear of crime did not moderate the effects of threat associations with Black men on any of the other policing policy items. This indicates that fear of crime only partially explains the relationship between threat associated with Black men and policing policy positions. Thus, it is possible that the relationship between Black threat and policing policy positions has more to do with racial bias than other intervening factors. Because the recent policing policy debate has been focused on racial disparities, those with higher racial bias (who may also feel threatened by Black people) may simply be less inclined to support policy reforms that are perceived to benefit Black people. This is consistent with Study 3 findings, in which those with lower prejudice (i.e., those with high IMS) were most supportive of policing policy reform.

## General discussion

Taken together, the results of the current work show that threat associations with police officers and threat associations with Black men reliably predict support for policing policy reform. As threat associated with police officers increases, participants are increasingly supportive of restrictive and reformed policing policies. Yet, as threat associated with Black men increases participants are increasingly resistant to restrictive and reformed policing policies. Furthermore, Study 3 provided evidence that experimentally manipulating threat associations with Black men can influence positions on policing policy. Participants with low racial bias (i.e., high IMS), who are typically supportive of policing policy reform, were significantly less supportive when exposed to threatening media images of Black men.

Since the Michael Brown shooting in August 2014, there has been a substantial public outcry over police use of excessive force, especially with Black men. This issue has also gained attention among psychologists who endeavor to identify solutions to the problem of excessive police force used on Black men (Hall et al., 2016). The current findings show that when it comes to policy decisions, such as whether police force demographics should match those of the communities they serve, associating Black men with threat significantly predicts positions. Those that associated Black men with threat tended to indicate that there were more instances in which lethal force was acceptable, were less inclined to scrutinize officers involved in incidents of deadly force, were less supportive of the use of on-body cameras, and were less supportive of demographic matching. These findings have important implications for public support for policing policy reform, such that those who feel threatened by Black men may be less likely to vote for reformed policing practices or candidates who support them. These findings may also have implications for how members of the public may perceive the appropriateness of police action in specific incidents of police use of force. Given that those who feel more threatened by Black men tend to want less scrutiny of officers involved in deadly force incidents, it is not unlikely that they would be less inclined to question an officers' actions in a specific incident, especially if the civilian involved in the incident were Black.

Furthermore, the current evidence indicates that exposing participants to threatening images of Black men can bias policing policy positions, such that associating Black men with threat may cause people to resist policing policy reform. The images used in Study 3 were drawn from online news sources' coverage of police protests—thus our findings suggest that the publication of such images in the media may have increased resistance to police reform, particularly among those that are high in IMS. The images used to prime police threat in Study 2 were also drawn from media coverage of police response to protests, yet only the threatening images of Black men (Study 3) influenced policing policy positions. Thus, the current findings suggest the public may be more susceptible to the effects of associating Black men with threat, relative to associating police officers with threat.

Overall, the subtle conditioning technique used to prime threat associations in Study 4 failed to produce the expected changes in policing policy positions. This suggests that perhaps the conditioning procedure may not have been strong enough to generate measurable changes in policing policy attitudes. Yet our measure of behavioral intentions did show movement in the expected direction. Participants' probability of signing a petition calling for police reform was significantly greater than chance in the control condition, whereas in the Black threat condition participants' willingness to sign the petition was at chance levels. Thus, Study 4 findings support the notion that priming associations between Black men and can threat influence behavioral intentions.

### Racial bias and fear of crime

Although the goal of the current research was not specifically to examine the motivational underpinnings of the relationship between threat associations and policing policy support, we did attempt to address this issue in Studies 3 and 4. There is a long history of racial prejudice against Black people in the U.S., thus it is quite possible that associations between Black men and threat are tapping into preexisting racial biases. Most Whites in the U.S. have at least some level of implicit bias against Black people (Nosek et al., 2002), which may predispose them to associate Black people with threat. In fact, recent evidence suggests that prejudice leads people to perceive threat, such that experimentally induced prejudice against unfamiliar groups leads to increases in threat associated with those groups (Bahns, 2015). Moreover, because the contemporary policing policy debate has been focused on racial disparities, those with higher racial bias may be less inclined to support policy reforms that are perceived to benefit Black people. The results of Study 3 provide some support for this notion, such that our proxy for racial bias (IMS) predicted support for policing policy reform. Specifically, as racial bias decreased (i.e., IMS increased) participants became more supportive of policing policy reform. Furthermore, we found that IMS moderated the effect of the Black threat prime. Under control conditions low prejudice participants (those with high IMS) were more supportive of policing policy reforms, yet when primed with Black threat low prejudice participants were no more supportive of policing policy reforms than high prejudice participants (those with low IMS). These findings suggest that racial biases may help explain the relationship between Black threat associations and policing policy positions.

Given the evidence from Study 3, that racial biases may help explain why Black-threat associations predict support for policing policy reform, Study 4 was designed to examine the mediating role of stereotypes. We predicted that because of stereotypes associating Black men with crime (Eberhardt et al., 2004), feeling threatened by Black men may result in a heightened fear of crime, which may then lead participants to want to give police officers more power to fight crime. We examined this possibility within our data finding that fear of crime only mediated support for one specific policy, demographic matching. Thus, overall threat associations were a much better predictor of policing policy position than fear of crime—such that when both were included in the models, fear of crime was not a significant predictor of policing policy positions. Another possible explanation for the association between feeling threatened by Black men and policing policy positions may be the contemporary cultural context, in which Black men (or Black people in general) and police are often seen as adversaries. In other words, within the current social context people may perceive Black men as a threat to police, therefore believing that we should not be restricting police power when they are routinely in conflict with such threatening adversaries. Yet, the weak and inconsistent relationship between the two threat association measures across samples, suggests that this is not the case.

Taken together the current findings suggest that racial biases may play an important role in the relationship between Black-threat associations and support for policing policy reform. The finding that priming associations between Black men and threat had some influence on policing policy positions, whereas priming associations between police and threat had none, suggests that these primes may be activating preexisting biases. There is robust evidence of prejudice against Blacks in the U.S. (Nosek et al., 2002), who are routinely stereotyped as hostile and aggressive (Devine and Elliot, 1995; Eberhardt et al., 2004). Previous findings indicate that automatic stereotype activation—such as the stereotype that Blacks are aggressive—leads to prejudiced responding (Devine, 1989). Although resistance to policing policy reform is not a measure of prejudice *per se*, it is certainly possible that stereotype activation leads to resistance to policy reforms that are perceived to benefit Blacks. Future research should further examine whether Black-threat associations can cause changes in policing policy position and the extent to which racial bias and stereotype activation explain the relationship between threat associations and policing policy position.

### Limitations and future directions

**Figure 3:**
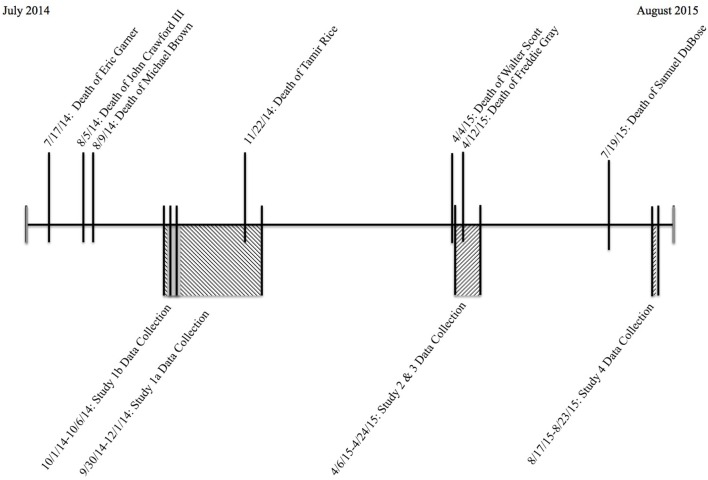
**Timeline of data collection for all studies within the context of critical events related to the policing policy reform debate**.

Across four studies we found that threat associations predicted support for policing policy reform. These findings show a general tendency to oppose reform and support less restrictive policing policies among those who associate Black men with threat. Associating police officers with threat predicted just the opposite. We suspect that variability in these relationships across samples on specific policy items may be a product of shifts in the national debate over these issues. All data were collected at a time in which extensive media coverage and debate surrounded the issue of race and policing policy reform, thus there could have been many ways in which the larger events happening in the real-world could have influenced our data. For example, while all data were collected after Michael Brown's death, amidst the Black Lives Matter movement, there continued to be highly publicized lethal encounters with police throughout the year of data collection (e.g., the deaths of Tamir Rice, Freddie Gray, Walter Scott, Samuel DuBose). See Figure 3 for a timeline of data collection for all studies within the context of critical events related to the policing policy reform debate. Thus, it may have been particularly challenging to experimentally manipulate threat associations in a cultural context in which threat associations are simultaneously being primed by the media. Intense media coverage and national debate over policing policy reform may have resulted in more intransigent policy positions. Future work, when debate over race and policing are not at the media forefront, may be able to address this issue.

Another limitation of the current study is that our sample was primarily composed of White participants. There is evidence that attitudes toward policing policy vary by participant race, such that Black Americans have less confidence in police and the criminal justice system than White Americans (Newport, 2014), thus it is unclear to what extent these findings would generalize to samples of Black Americans or members of other racial minority groups. Although controlling for participant race and ethnicity left results largely unchanged, additional research is needed to confirm that these findings do not vary across racial groups. Another consideration is that three of our five samples were made up of young college students, who tend to have the least confidence in police of all age groups (Jones, 2005), although this generalization may not apply to the fairly conservative students that participated in our studies. Indeed, there is evidence that findings established in college samples do not always generalize to the larger population (Peterson, 2001; Henrich et al., 2010), thus these results should be viewed with some caution. Study 1a findings (college sample) were all replicated in Study 1b (adult community member sample), providing some evidence that our findings can be generalized beyond college students. Yet, replication of this research in larger and more diverse community samples will be critical in determining the generalizability of these findings.

## Conclusions

Racial disparities in policing are well known to social scientists (Goff and Kahn, 2012). Traffic stops and “stop and frisk” policies in the United States disproportionately target Black and Latino men (Walker et al., 2011). Relative to Whites, racial minorities are significantly more likely to have police use force against them (Walker et al., 2011; Bolger, 2014). Efforts to remedy these issues show promise. Racially diverse police departments tend to be more democratic and may have more credibility within racial minority communities (Sklansky, 2006). Yet, racial minorities are still vastly underrepresented in major metropolitan police departments throughout the United States (Ashkenas and Park, 2014). For example, in 2014 although the population of Ferguson, Missouri, was 67% Black, less than 6% of the police force was Black (Lowery et al., 2014). Identifying potential barriers to such reforms, such as perceived threat associated with Black men, may be the first step in addressing these issues and moving toward reform. Moreover, recognizing the existence of such biases may have important implications for policing policies, as well as perceptions of specific incidences, such as those covered by Kingsley v. Hendrickson (2015).

Our findings suggest that publicizing racially charged police encounters may actually promote *resistance* to policing policy reform among those who perceive Black men as threatening. This is particularly important given that protests designed to draw attention to incidents of excessive force may inadvertently increase threat associated with Black men, leading to resistance to policing policy reform. This is consistent with recent research indicating that emphasizing racial inequality in the prison system might actually increase support for injustice enforcing policies (Hetey and Eberhardt, 2014). Thus, for those looking to reform policing policies, emphasizing the problems with police use of force across demographic groups and ensuring that police protests remain nonviolent may be critical in increasing support for policing policy reform.

## Author contributions

All authors contributed to the development of the study concept and study design. AS performed the data analysis and interpretation. AS drafted the manuscript, and IH provided critical revisions. Both authors approved the final version of the manuscript for submission.

## Funding

This work was supported by the Society for the Psychological Study of Social Issues (Grant-in-Aid to IH).

### Conflict of interest statement

The authors declare that the research was conducted in the absence of any commercial or financial relationships that could be construed as a potential conflict of interest.

## Footnotes

^1^IAPS codes were: 1026, 1050, 1114, 1120, 1200, 1205, 1220, 1300, 1302, 1304, 1321, 1440, 1460, 1463, 1525, 1595, 1731, 1820, 1850, 1910, 1930, 1932, 2002, 2019, 2034, 2035, 2036, 2040, 2091, 2156, 2165, 2222, 2302, 2362, 2390, 2489, 2575, 2616, 2791, 5001, 5250, 5390, 5395, 5470, 5535, 5623, 5660, 5711, 5731, 5825, 5833, 5891, 7013, 7160, 7161, 7325, 7450, 7530, 8001, 8241, 8503, 8510, 8531, 8540, 9210, 9260, 9360, 9468, 9469, 9831.

^2^Results from the model controlling for participant demographic factors was similar, yet including demographic factors in the model substantially increased standard errors such that none of the probabilities significantly differed from chance (Black threat condition: *Probability* = 0.56, *SE* = 0.12, *p* = 0.625; police threat condition: *Probability* = 0.63, *SE* = 0.11, *p* = 0.243; control threat condition: *Probability* = 0.68,*SE* = 0.11, *p* = 0.113). Given the increase in error introduced by the demographic control variables we are hesitant to draw conclusions from the differences in statistical significance across models.
