# Supplementary material for: Perceived Threat Associated with Police Officers and Black Men Predicts Support for Policing Policy Reform
Source: Front Psychol. 2016 Jul 12;7:1057. doi: 10.3389/fpsyg.2016.01057 (PMC4940419; doi:10.3389/fpsyg.2016.01057)
Supplement: Supplementary file 1 [file Table1.DOCX]

SUPPLEMENTAL MATERIALS

*Table S1*. Table represents the percentage of participants responding in the affirmative to “when do you believe it is appropriate for law enforcement officers to use deadly force?” in each circumstance in Study 1b.

| When someone is using deadly force on an officer | 93.30% |
| --- | --- |
| When someone is physically injuring an officer | 54.60% |
| When an officer believes someone will use deadly force on them | 50.20% |
| When an officer believes someone will physically injure them | 17.80% |
| When someone is committing a crime. | 11.90% |
| If someone has committed a crime | 7.40% |
| When someone is non-cooperative | 5.90% |
| When an officer believes someone will be non-cooperative | 1.10% |
| When an officer believes someone will be committing a crime | 0.70% |

*Table S2*. Table represents the percentage of participants responding in the affirmative to “when do you believe it is appropriate for law enforcement officers to use deadly force?” in each circumstance in Study 2.

| When someone is using deadly force on an officer | 86.70% |
| --- | --- |
| When someone is physically injuring an officer | 59.20% |
| When an officer believes someone will use deadly force on them | 48.00% |
| When someone is committing a crime. | 17.30% |
| When an officer believes someone will physically injure them | 13.30% |
| If someone has committed a crime | 2.00% |
| When someone is non-cooperative | 3.10% |
| When an officer believes someone will be committing a crime | 1.00% |
| When an officer believes someone will be non-cooperative | 2.00% |

*Table S3*. Table represents the percentage of participants responding in the affirmative to “when do you believe it is appropriate for law enforcement officers to use deadly force?” in each circumstance in Study 3.

| When someone is using deadly force on an officer | 89.10% |
| --- | --- |
| When someone is physically injuring an officer | 54.70% |
| When an officer believes someone will use deadly force on them | 51.60% |
| When someone is committing a crime. | 17.20% |
| When an officer believes someone will physically injure them | 14.10% |
| If someone has committed a crime | 6.30% |
| When someone is non-cooperative | 3.90% |
| When an officer believes someone will be committing a crime | 0.80% |
| When an officer believes someone will be non-cooperative | 0.80% |

*Table S4*. Table represents the percentage of participants responding in the affirmative to “when do you believe it is appropriate for law enforcement officers to use deadly force?” in each circumstance in Study 4.

| When someone is using deadly force on an officer | 97.20% |
| --- | --- |
| When someone is physically injuring an officer | 50.50% |
| When an officer believes someone will use deadly force on them | 52.80% |
| When someone is committing a crime. | 7.50% |
| When an officer believes someone will physically injure them | 17.00% |
| If someone has committed a crime | 6.10% |
| When someone is non-cooperative | 3.80% |
| When an officer believes someone will be committing a crime | 1.90% |
| When an officer believes someone will be non-cooperative | 0.90% |
